# Cumulative IgE-levels specific for respiratory allergens as biomarker to predict efficacy of anti-IgE-based treatment of severe asthma

**DOI:** 10.3389/fimmu.2022.941492

**Published:** 2022-09-21

**Authors:** Veronika Naumova, Evgeny Beltyukov, Katarzyna Niespodziana, Peter Errhalt, Rudolf Valenta, Alexander Karaulov, Darina Kiseleva

**Affiliations:** ^1^Department of Faculty Therapy, Endocrinology, Allergology and Immunology, Ural State Medical University, Ekaterinburg, Russia; ^2^Department of Pathophysiology and Allergy Research, Center for Pathophysiology, Infectiology and Immunology, Medical University of Vienna, Vienna, Austria; ^3^Department of Pneumology, University Hospital Krems and Karl Landsteiner University of Health Sciences, Krems, Austria; ^4^Karl Landsteiner University of Health Sciences, Krems, Austria; ^5^Laboratory of Immunopathology, Department of Clinical Immunology and Allergy, Sechenov First Moscow State Medical University, Moscow, Russia; ^6^National Research Centre (NCR) Institute of Immunology Federal Medical-Biological Agency (FMBA) of Russia, Moscow, Russia

**Keywords:** asthma biomarker, Omalizumab, biologicals, molecular therapy, targeted therapy

## Abstract

Molecular therapies, including anti-IgE, biologicals and small molecules are increasingly used for treatment of asthma. The effectiveness of these therapies may be increased with biomarkers. Aim of this study was to assess the value of measuring cumulative IgE levels specific for respiratory allergens to increase the efficacy of anti-IgE therapy for severe bronchial asthma. One hundred and thirty seven patients with severe asthma were recruited from 2016 to 2022. Standard empirical allergy diagnosis (i.e., anamnesis, skin testing, allergen-specific IgE measurement), blood eosinophil counting, measurement of total IgE and of cumulative IgE-specific for respiratory allergens by Phadiatop™ were performed. Thirty four patients with severe allergic asthma, for whom all three diagnostic methods were performed, were then used to analyze the efficacy of anti-IgE treatment in patients stratified in two groups according to cumulative IgE levels specific for respiratory allergens determined by Phadiatop™. Group #1 patients (n = 8) had cumulative specific IgE values ≥ 0.35 and < 1.53 PAU/l while in group #2 patients (n = 26) they were ≥ 1.53 PAU/l. Treatment with Omalizumab was performed for at least 12 months. The level of asthma control (ACT questionnaire), the number of asthma exacerbations, the quality of life (AQLQ questionnaire), the need for systemic corticosteroids, and the respiratory function (FEV1) was determined by “before-after” analysis for each group, followed by a comparison of the dynamics between groups. In group 2 patients with an initial allergen-specific IgE level ≥ 1.53 kUA/L, the efficacy of Omalizumab treatment was better regarding asthma control, number of exacerbations, and quality of life than in group 1 patients. Our study provides evidence that measuring cumulative levels of IgE specific for respiratory allergens could be a useful screening method for detecting an allergic phenotype of severe asthma and may serve as biomarker to enhance the success of IgE-targeted therapy.

## Introduction

New opportunities have appeared in the treatment of severe bronchial asthma due to the development of targeted molecular therapies (1, 2). However, the effectiveness of molecular therapies depends on the correct diagnosis of the disease phenotype and the adequate drug selection (3–6). Accordingly, many researchers are looking for biomarkers for various asthma phenotypes (7–13). These biomarkers should be accurate, available in practice and cost-effective. To determine the allergic phenotype of asthma, it is important to prove clinically the presence of a clinically relevant allergen-specific IgE sensitization. This can be achieved by comparing allergic anamnesis and the results of standard diagnostic methods which are usually selected empirically (skin testing and/or measurement of specific IgE and/or allergen provocation testing) and/or anamnesis in combination with comprehensive assessment of multiple IgE sensitizations (7, 14–17). Skin tests are usually reliable, relatively inexpensive but do not necessarily prove the presence of allergen-specific IgE, while provocation tests are rarely used in patients with severe asthma because of the risk of severe systemic reactions. Furthermore, these methods are particularly performed with allergen extracts prepared from the natural allergen sources and thus represent mixtures of allergenic and non-allergenic substances. Although extract-based tests can provide information about the sensitizing allergen source, they do not inform to which specific allergenic component(s) present in the source the patient is sensitized to (18). Therefore, defined allergen components have been introduced in the diagnosis of allergy and different component-based tests have been developed to measure allergen-specific IgE levels in blood of a patient as biomarkers for a clinically relevant allergen exposure associated with symptoms of allergy (17, 19–21). Determination of allergen-specific IgE by *in vitro* methods is safe for patients but testing with single allergen molecules or micro-arrayed allergens might be expensive (17). One inexpensive possibility for simultaneous testing of IgE sensitizations to multiple respiratory allergen sources is the Phadiatop^ТМ^ test, which has been studied as a screening method for detecting allergen-specific IgE sensitization from the late 1980s (22–29) to the presence (30, 31). This test is based on simultaneous testing for serum specific IgE to a mixture of allergens causing common inhalant allergies. The high value of Phadiatop™ was evaluated in the general population to identify patients with allergic sensitizations (28, 29) among patients with symptoms of airway obstruction (26) and among children with suspected respiratory allergies, (32) among adults with asthma (30, 33) and children with asthma (34) among patients with allergic rhinitis (27, 31, 35), among patients with asthma and/or rhinitis (23, 24, 36–39). In these studies the Phadiatop^ТМ^ showed a sensitivity of 70% (29) to 100% (25, 33) and a specificity of 83% (33) to 100% (23, 26). The ineffectiveness of measuring total IgE for the precise diagnosis of allergic diseases has been shown in several studies (23, 26, 28, 31).

Today, patients with uncontrolled severe asthma are routinely considered as candidates for molecular therapies, which target specific inflammatory pathways involved in the pathogenesis of asthma (40). Omalizumab, a therapeutic anti-IgE antibody, is the first globally approved and most often used targeted molecular therapy of severe or moderate to severe allergic asthma. It is administered subcutaneously every two or four weeks at a dose determined according to the patient’s body weight and serum total IgE levels (30 – 1500 IU/mL), ranging from 75 to 600 mg. Clinical studies performed with Omalizumab in the last 10 years have confirmed its effectiveness and safety in the treatment of severe asthma by reducing symptoms, frequency of reliever use, and severe exacerbations but there are also non-responders to this treatment (41).

Accordingly, there is an unmet need for biomarkers which are useful for identifying patients suffering from allergic asthma and which at the same time can be used to predict the efficacy of anti-IgE treatment (42, 43). The measurement of total IgE levels has been suggested as one possible biomarker (42) but there seem to be no clear cut-off levels and it even was suggested that subjects with low total IgE levels benefit most from treatment (44). Another study performed a retrospective investigation of poly- versus monosensitized patients but the results were not clear (45). Yet another study demonstrated that the proportion of allergen-specific IgE antibodies in relation to total IgE may be useful to predict the efficacy of anti-IgE treatment indicating the importance of the measurement of allergen-specific IgE levels (46). However, two studies provided counter-intuitive information. One study found no relation between pretreatment specific IgE and response to treatment (47) and another study even showed that Omalizumab is also effective in non-atopic asthma (48).

We hypothesized that the measurement of the cumulative amount of IgE specific for respiratory allergens may be useful to identify patients suffering from severe allergic asthma and eventually may even serve as a biomarker to predict the efficacy of anti-IgE treatment.

## Materials and methods

### Patients, study design and ethical considerations

One hundred and thirty seven adult patients, who were registered in the Sverdlovsk region, Russia from 2016 to April 2022, were enrolled in this study. Inclusion criteria included patients (18 years and older) with severe bronchial asthma, who were diagnosed according to the ATS/ERS criteria from 2014 (49) and GINA guidelines (50, 51). Asthma was considered as not controlled by GINA steps 4-5 (moderate or high ICS with a second baseline drug, continuous SGCS), or by the fact that this treatment was required to maintain control and to reduce the risk of exacerbations. Exclusion criteria included confirmed helminthic invasion, cytostatic therapy, pregnancy, severe somatic pathology (heart failure 3-4 functional classes, liver failure according to Child-Pugh class B and C, chronic kidney disease C3a and above) and the inability to meet the schedule of visits for injection, evaluation of efficacy and safety. All subjects provided their written informed consent. The study was approved by the ethics committee of the Ural State Medical University and conducted according to the Declaration of Helsinki.

### Diagnostic methods

Allergic phenotype was determined based on standard diagnostic methods for allergy including anamnesis, skin prick tests (52), total and specific IgE determination according to a traditional pathway (53), and Phadiatop™ ImmunoCAP measurements of IgE specific for a mix of inhalant allergen sources (hereinafter referred to as Phadiatop™). An allergic phenotype was defined by a combination of a positive allergic anamnesis and positive skin testresult or a positive allergic anamnesis and at least one positive specific IgE. An eosinophilic phenotype of bronchial asthma was defined in the case of negative allergic anamnesis, the absence of proven clinically relevant sensitization, and a concentration of eosinophils exceeding 150 cells/μL. The mixed phenotype of asthma was defined as a combination of a positive allergic anamnesis, late onset of asthma, eosinophilia of more than 300 cells/μL.

### Anamnesis and skin testing

Anamnesis was assessed in a comprehensive manner according to the presence of a clinically relevant reaction of the patient (symptoms of asthma, rhinoconjunctivitis, urticaria and angioedema) upon contact with an allergen source, the effect of elimination of exposure and/or the presence of relatives suffering from allergic diseases. Skin tests were assessed retrospectively if patients had earlier positive skin test reactions specific for allergen sources (house dust, house dust mites, cat, dog, a mixture of trees, meadow grasses, weeds) with positive (histamine) and negative (saline) controls (52).

### Determination of total and allergen-specific IgE levels

Determination of total IgE was performed using a chemiluminescent method (IMMULITE 2000, SIEMENS, Erlangen, Germany). Total IgE was considered as elevated at a level ≥ 100 IU/ml. Specific IgE was determined by the ImmunoCAP method (immunofluorescence on a three-dimensional porous solid phase, Phadia 250, Phadia) (Thermofisher, Uppsala, Sweden) to dog dandruff allergens (Dog dander e5), house dust mite allergens (*Dermatophagoides pteronyssinus* D1, *Dermatophagoides farinae* D2), epithelium of a cat (Cat Dander-Epithelium E1), house dust (Greer H1), mold (*Penicillum notatum* M1), birch pollen (Birch T3), a mixture of grass allergens (Orchard Grass, Meadow Fescue, Perennial Rye Grass, Timothy Grass, June Grass (Kentucky Blue) GP1), mugwort pollen (*Artemisia vulgaris* W6). A result was considered positive when specific IgE levels were ≥ 0.35 kUA/l. Phadiatop™ was performed using the ImmunoCAP technique (immunofluorescence on three-dimensional porous solid phase, Phadia 250, Phadia). Phadiatop™ level ≥ 0.35 PAU/l was reported as positive result, and < 0.35 PAU/l as negative result.

### Evaluation of diagnostic methods and the efficacy of Omalizumab treatment

Sensitivity, specificity, accuracy, positive and negative predictive values of diagnostic methods were determined in four-field tables separately for each method and in combinations. For this analysis patients with severe allergic and non-allergic asthma were studied, in whom all 3 methods had been performed (i.e., taking allergic anamnesis, standard diagnostic methods such as skin testing and/or measuring allergen-specific IgE and/or Phadiatop™ testing). Patients with mixed bronchial asthma (n = 4) were not included in the analysis. Patients with the diagnosis of severe allergic asthma were selected for the treatment with Omalizumab (Novartis, Basel, Switzerland) for at least 12 months according to the instructions of the manufacturer, taking into account the patient’s body weight and the level of total IgE (the corresponding amount of mg of the drug 1 or 2 times a month). Each patient had evaluation visits before starting the treatment with Omalizumab (baseline), at 4 months of therapy, and at 12 months of therapy. At each of these visits, patients completed the ACT (Asthma Control Test), AQLQ (Asthma Quality of Life Questionnaire) questionnaires, spirometry (=respiratory function) was performed, and data on exacerbations were collected. Spirometry was done according to ATS/ERS recommendations 2005 and GINA guidelines (54). Exacerbation of asthma was defined as the worsening of symptoms requiring an increase in of therapy (increasing the dose of inhaled corticosteroids, prescribing systemic corticosteroids or increasing the dose of systemic corticosteroids) and/or hospitalization. Patients were asked about the number of exacerbations in the year before baseline and between visits after the initiation of Omalizumab therapy.

### Statistical analysis

Statistical analysis was performed using StatTech v. 2.1.0 (Developer - StatTech LLC, Russia), NCSS Statistical Software 2021 (https://www.ncss.com/). Quantitative variables were assessed for normality using the Shapiro-Wilk test or the Kolmogorov-Smirnov test. Quantitative variables following a normal distribution were described using mean (M) and standard deviation (SD), 95% confidence interval (95% CI) for the mean were estimated. Quantitative variables following the non-normal distribution were described using median (Me) and lower and upper quartiles (Q1 – Q3). Student’s t-test or Mann-Whitney-U test were used for comparisons between two groups following the normal or non-normal distribution of the data, respectively. Comparisons of three or more groups were done using the Kruskal-Wallis test and Dunn’s criterion with Holm correction as a *post-hoc* method. P-value under the set up value of α = 0.05 (two-sided) was considered as statistically significant. ROC analysis was used to assess the diagnostic performance of quantitative variables in predicting a categorical outcome. The optimal cut-off value was estimated using the Youden’s index. Comparison of frequencies in the analysis of 2 by 2 contingency tables was performed using Pearson’s chi-square test (for expected values greater than 10), and Fisher’s exact test (for expected values less than 10). One-way repeated measures analysis of variance was used to compare three or more matched samples in regard to normally distributed quantitative variables. Statistical significance of dependent variable changes over time was assessed using the Pillai’s Trace and paired Student’s t-test with Holm correction as *post hoc* methods. When comparing three or more matched samples regarding to non-normally distributed quantitative variables Friedman test was used along with ConoverIman test with Holm correction as a *post hoc* method.

## Results

### Cumulative specific IgE levels measured by Phadiatop™ are useful to determine phenotypes of severe asthma

**Figure 1:**
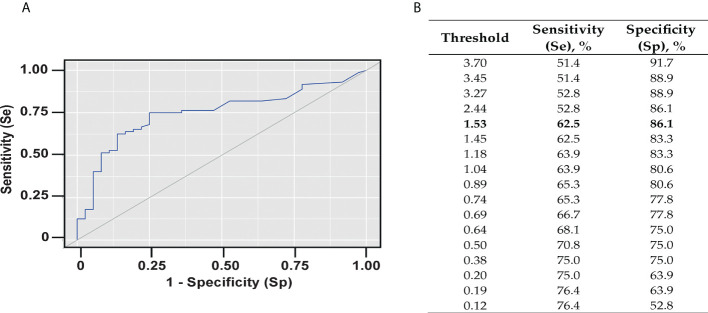
Diagnostic performance of Phadiatop™ test. **(A)** ROC-curve characterizing the probability of a positive allergic anamnesis depending on the results of the Phadiatop™ test. **(B)** Threshold values of the Phadiatop™ test. Sensitivity (Se) is defined as the proportion of positive tests among atopics. Specificity (Sp) is the percentage of negative tests among non-atopics.

This study evaluated a total number of 137 subjects with severe bronchial asthma, who were included in the regional register of Sverdlovsk region from 2016 to April 2022. **Table 1** shows a detailed characterization of these patients and the diagnostic methods performed in real clinical practice. According to the International Classification of Diseases (ICD), patients with severe asthma (SA) were divided into three groups: Allergic SA (J45.0; n = 57), non-allergic SA (J45.1; n = 61), and mixed SA (J45.8; n = 19). The median age of the participants was 52 years (range: 43 – 59) . One hundred and fourteen (i.e., 83.2%) were women, twenty three (i.e., 16.8%) were men and fifty seven (i.e., 41.6%) had an allergic phenotype of severe asthma. Phadiatop™ tests were performed in 107 (i.e., 78.1%) of the patients. The diagnostic methods performed in the patients are shown in **Table 1**. Phadiatop™ test results and total IgE levels for the severe asthma patients are shown in **Table 2**. Cumulative IgE levels specific for respiratory allergens as determined with Phadiatop™ testing in patients with allergic and mixed severe asthma were significantly higher than those in patients with non-allergic asthma (**p < 0.001). The same dynamics was detected for total IgE level (*p = 0.002) (**Table 2**). Data obtained by Phadiatop™ testing were then compared with the results of standard diagnostic methods for determining the allergic status of asthmatic patients. In groups of patients with a positive allergic anamnesis, positive skin tests or positive allergen-specific IgE obtained by standard empirical testing, specific IgE levels determined by the Phadiatop™ test were significantly higher than in the groups with negative results obtained by each of the three standard allergy test methods (**Table 3**). By contrast, total IgE levels in the groups with positive and negative allergic anamnesis, positive and negative skin test results did not differ significantly from each other. Statistically significant differences in regard to total IgE levels were only obtained for the groups with negative and positive allergen-specific IgE (*p = 0.047) (**Table 3**). To assess the diagnostic performance of Phadiatop™ test results for predicting a positive allergic anamnesis a ROC-curve analysis was performed (**Figure 1A**). The calculated area under the ROC curve comprised 0.750 ± 0.047 with 95% CI: 0.658 - 0.841 and the resulting model was statistically significant (**p < 0.001). A cut-off value of specific IgE levels (i.e., 1.530 PAU/l) measured by Phadiatop™ test was also determined and found to correspond to the highest Youden’s index **(** **Figure 1B**). If the cumulative IgE specific for respiratory allergens determined by Phadiatop™ was greater than or equal to 1.530 PAU/l, it predicted the presence of a positive allergic anamnesis. The sensitivity and specificity of the method was 62.5% and 86.1%, respectively (**Figure 1A**). In addition, if specific IgE levels determined by the Phadiatop™ test were ≥ 0.380 PAU/l positive skin test results were predicted with a sensitivity of 81.8% and with a specificity of 90.5%. If specific IgE levels measured by Phadiatop™ were ≥ 0.380 PAU/l the presence of specific IgE as determined by empirical individual testing of specific IgE was predicted with 90.0% sensitivity and 77.8% specificity. Furthermore, a comparison of different diagnostic methods was performed separately and in combinations depending on the phenotype of asthma (**Table 4**). Measurement of specific IgE by Phadiatop™ showed a sensitivity of 94.44%, which was the same as the sensitivity of allergic anamnesis and inferior to the standard empirical diagnostic methods, which showed 100% sensitivity (**Table 4**). However, the specificity of 83.78% of the Phadiatop™ test turned out to be higher than the specificity of allergic anamnesis and standard diagnostic methods, showing 64.86% and 62.16% of specificity, respectively (**Table 4**). The best results were obtained when Phadiatop™ testing was combined with allergic anamnesis (sensitivity 100%, specificity 87.5%, accuracy 94.64%, PPV 91.43%, NPV 100%) (**Table 4**).

**Table 1:** Characteristics of the patients and examination methods.

Characteristic	Allergic asthma	Non-allergic asthma	Mixed asthma	Total
Women, n (%)	44	54	16	**114** (83,2)
Men, n (%)	13	7	3	**23** (16,8)
Age, Me (Q1-Q3)	46 (38–54)	57 (50–62)	51 (42–58)	**52 (43-59)**
Allergic anamnesis	57	61	19	**137**
Standard methods (skin tests and/or sIgE)	49	46	16	**111**
Phadiatop™	42	48	19	**107**
Total IgE	52	51	18	**121**
Allergic anamnesis + Phadiatop™ + Standard methods	36	37	15	**88**
Allergic anamnesis + Standard methods	49	46	16	**111**
Allergic anamnesis + Phadiatop™	42	48	17	**107**

**Table 2:** Specific IgE measured by Phadiatop™ and total IgE levels in patients with different phenotypes of severe asthma.

Severe asthma phenotype		Phadiatop™ (PAU/l)			^1^p	Total IgE (IU/ml)			^1^p
		Me	Q^1^ – Q^3^	n		Me	Q^1^ – Q^3^	n
Allergic asthma		5.23	1.67 – 11.12	42		199.8	97.8 – 396.2	52
Non-allergic asthma		0.08	0.02 – 0.15	49	< 0.001	96.0	29.9 – 204.5	51	0.002
Mixed asthma		7.10	3.64 – 11.93	18		242.4	117.8 – 890.0	18

^1^differences are statistically significant (**p < 0.001).

**Table 3:** Phadiatop™ test results and standard diagnostic methods in different phenotypes of severe asthma.

Standard diagnostic method		Phadiatop™ (PAU/l)			^1^p	Total IgE (IU/ml)			^1^p
		Me	Q^1^ – Q^3^	n		Me	Q^1^ – Q^3^	n
Allergic anamnesis	negative allergic anamnesis	0.10	0.04 – 0.42	36	< 0.001	133.0	49.7 – 351.5	41	0.218
	positive allergic anamnesis	3.78	0.33 – 10.30	72		165.0	91.0 – 395.5	79
Skin tests	negative skin tests	0.09	0.04 – 0.17	21	< 0.001	122.0	52.2 – 216.9	22	0.100
	positive skin tests	4.45	0.79 – 10.30	55		190.3	95.5 – 421.0	60
Specific IgE	negative sIgE	0.04	0.01 – 0.31	9	< 0.001	129.0	42.0 – 194.5	11	0.047
	positive sIgE	3.36	0.93 – 8.17	30		277.3	96.2 – 604.0	32

^1^differences are statistically significant (*p < 0.05; **p < 0.001).

**Table 4:** Characteristics of diagnostic methods for defining allergic asthma and their combinations.

	Phadiatop™		Anamnesis		Standard methods (skin tests and sIgE)		Anamnesis + Phadiatop™		Anamnesis + standard methods	
	+	-	+	-	+	-	+	-	+	-
**Allergic asthma**	34	2	34	2	35	1	32	0	34	1
**Non-allergic asthma**	6	31	13	24	14	23	3	21	11	12
**Sensitivity**	94.44% (81.34-99.32%)		94.44% (81.34-99.32%)		97.22% (85.47-99.93%)		100% (89.11-100.00%)		97.14% (85.08-99.93%)	
Specificity	83.78% (67.99-93.81%)		64.86% (47.46-79.79%)		62.16% (44.76-77.54%)		87.5% (67.64-97.34%)		65.62% (46.81-81.43%)	
Accuracy	89.04% (79.54-95.15%)		79.45% (68.38-88.02%)		79.45% (68.38-88.02%)		94.64% (85.13-98.88%)		82.09% (70.80-90.39%)	
Positive predictive value	85.0% (73.06-92.21%)		72.34% (62.63-80.32%)		71.43% (62.24-79.13%)		91.43% (78.73-96.85%)		75.56% (65.62-83.35%)	
Negative predictive value	93.94% (80.00-98.36%)		92.31% (75.35-97.92%)		95.83% (76.61-99.38%)		100%		95.45% (74.96-99.33%)	

### Allergen-specific IgE levels may predict the efficacy of Omalizumab therapy

Next, we investigated if the measurement of cumulative IgE values specific for respiratory allergens by Phadiatop™ can be useful to predict the efficacy of anti-IgE treatment in patients with severe asthma. The analysis included 34 patients with severe allergic asthma (J45.0) treated with Omalizumab for at least 12 months for whom all three diagnostic methods had been performed. The patients were divided into two groups. Group #1 (n = 8) included patients with cumulative IgE levels to respiratory allergens ≥ 0.35 and < 1.53 PAU/l while group #2 (n = 26) included patients with specific IgE levels ≥ 1.53 PAU/l. The efficacy of Omalizumab treatment was assessed by the level of asthma control (ACT questionnaire), the number of asthma exacerbations within one year before and one year after the initiation of therapy, the quality of life (AQLQ questionnaire), the need for systemic corticosteroids, and the respiratory function (FEV1). Patients were examined before the start of therapy, and after 4 and 12 months of therapy. “Before-after” analysis was performed for each group, followed by a comparison of the dynamics between groups.

**Figure 2:**
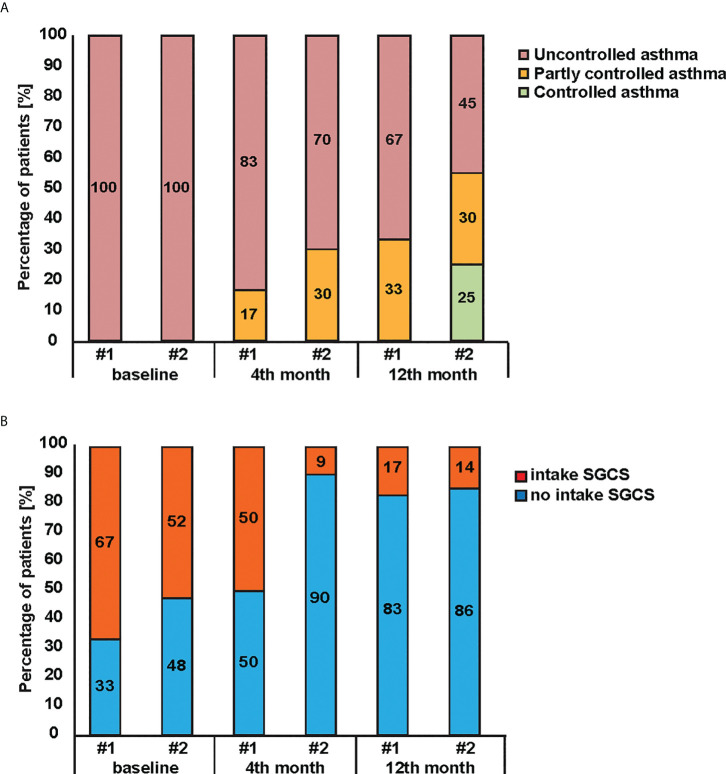
Effectiveness of Omalizumab therapy in patients initially diagnosed with Phadiatop™ test. Changes (y-axes) of **(A)** asthma control levels and **(B)** systemic glucocorticosteroids intake during Omalizumab therapy in patients with initial Phadiatop™ test value < 1.53 PAU/l (i.e. group #1) and > 1.53 PAU/l (i.e. group #2).

**Figure 3:**
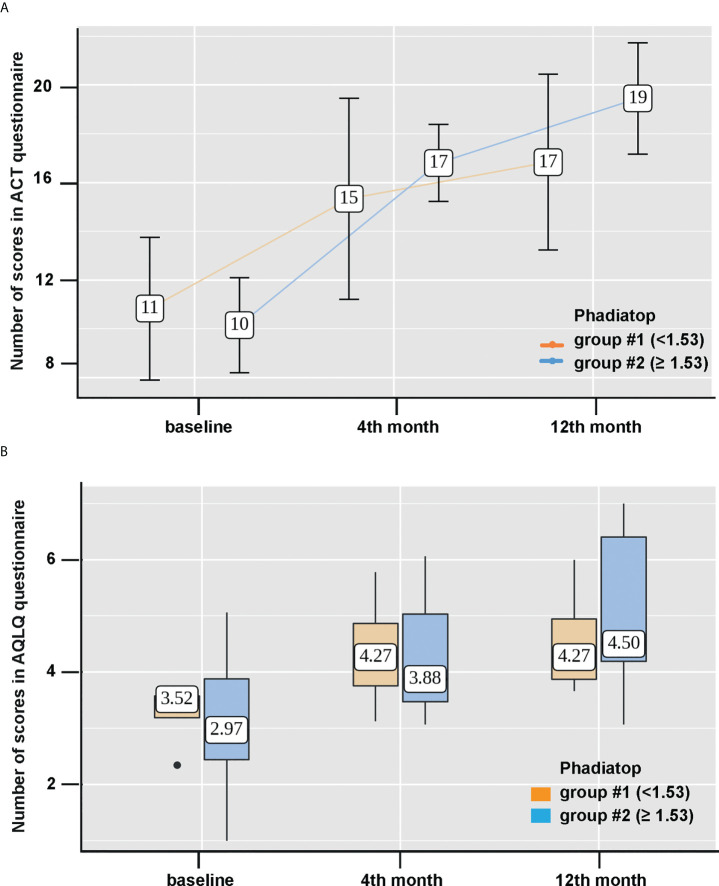
Improvement of asthma control level and the quality of life after Omalizumab treatment. **(A)** Changes of asthma control levels according to ACT questionnaire scores (points) and **(B)** dynamics of the quality of life according to AQLQ questionnaire scores (points) during Omalizumab therapy based on the initial value of the Phadiatop™ test (y-axes). Group 1, < 1.53 PAU/l (light brown, left) and group 2, > 1.53 PAU/l (blue, right) were compared. #stands for number in **Figure 3**.

The analysis of Omalizumab therapy efficacy in group #1 (Phadiatop™ test < 1.53 PAU/l) and group #2 (Phadiatop™ test ≥ 1.53 PAU/l) revealed a significant decrease in the proportion of uncontrolled bronchial asthma in both groups (*p = 0.018 and **p < 0.001, respectively) (**Figure 2A**). The reduction of uncontrolled asthma was more pronounced in group #2 where asthma was controlled in 26% of the patients (**Figure 2A**) However, the difference in asthma control levels between the groups observed after 12 months of therapy was not statistically significant (**Figure 3A**). Prior to the start of IgE-targeting therapy, 66.7% of patients in group #1 and 52.4% of patients in group #2, respectively, were taking systemic glucocorticosteroids (**Figure 2B**). After 4 months only 9% of patients in group #2 took systemic corticosteroids as compared to 50% in patients of group #1. After month 12 of Omalizumab therapy 83.3% of patients in group #1 and 85.7% of patients in group #2 did not require systemic glucocorticosteroids (p = 0.097 and **p < 0.001, respectively). There were no statistically significant differences in the dynamics between the groups (**Figure 2B**).

Then, we assessed the scores on the ACT questionnaire in both groups and the dynamics of the results was statistically significant from 11 (Q1-Q3: 8-14) to 17 (Q1-Q3: 13-20) points in group #1 and from 10 (Q1-Q3: 8 -12) to 19 (Q1-Q3: 17-22) points in group #2 (p = 0.015 and **p < 0.001, respectively) (**Figure 3A**). However, the changes were more pronounced in group #2, starting from month 4 of the therapy (**Figure 3A**). A significant improvement in the quality of life according to AQLQ questionnaire for a year of Omalizumab therapy was also observed in both groups (p = 0.039 and **p < 0.001). However, between the groups the differences by 12 months of therapy were not statistically significant (**Figure 3B**).

**Figure 4:**
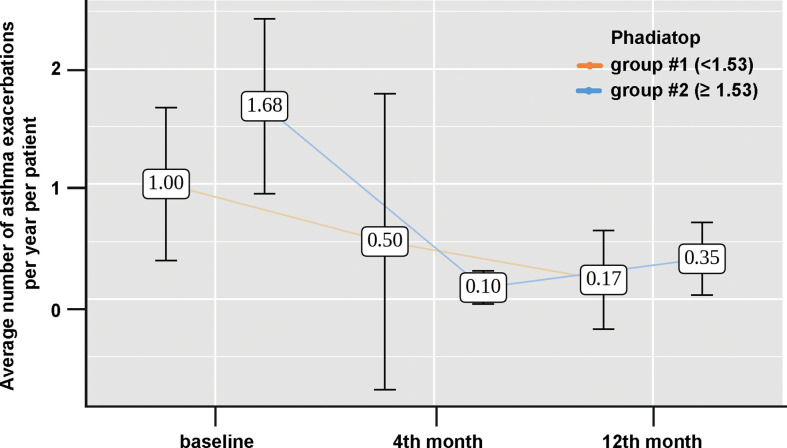
Changes of the number of asthma exacerbations during Omalizumab therapy depending on the initial value of the Phadiatop™ test (Group 1, < 1.53 PAU/l light brown and group 2, > 1.53 PAU/l blue).

In addition, we also observed a decrease in the number of exacerbations from 1.68 initially to 0.35 at month 12 of therapy (**p < 0.001) in group #2 while in group #1 there was no significant reduction in the number of asthma exacerbations per year per patient of Omalizumab therapy (p = 0.277) (**Figure 4**).

Finally, we also assessed respiratory function by FEV1. Group #1 showed an increase from 46% at baseline (95% CI 44.9 – 52.2) to 59.1% (95% CI 56.0 – 62.8) in month 12 (p = 0.311). Group #2 also had an increase from 69% (95% CI 48.0 – 77.1) at baseline to 78% (95% CI 56.0 – 94.0) after 12 months (p = 0.0233). Regarding FEV1 there were no statistically significant differences between the groups (data not shown).

## Discussion

Molecular testing using defined recombinant allergen molecules has revolutionized the understanding and diagnosis of allergy (17, 53). Serological tests containing a broad panel of allergen molecules have been developed to discriminate subjects with and without IgE sensitizations and to support accurate prescription of targeted immunotherapies (55, 56). It has been suggested that the assessment of allergic sensitization and antibodies specific for respiratory viruses, in particular to rhinovirus can be useful to identify forms of asthma triggered by allergen exposure and/or infection by respiratory viruses for personalized asthma treatment according to the responsible trigger factors (21, 55). Determination of virus-triggered forms of asthma can be achieved with chips containing micro-arrayed viral proteins and/or peptides which allow measuring increases of virus-specific antibody levels after the asthma attack or by measuring cumulative virus-specific antibody levels (57, 58). For the assessment of allergic sensitization tests are needed which allow to assess IgE sensitization to a broad panel of allergen molecules or alternatively by using a simple screening test combining allergens from a large panel of allergen sources such as the Phadiatop™ test (29, 30, 59). Previous studies have investigated the sensitivity and specificity of the Phadiatop™ test among the general population, among patients with allergic rhinitis and/or bronchial asthma to detect allergic IgE sensitization (23, 26, 27, 30–39). However, we did not find studies which have evaluated the usefulness of the measurement of cumulative IgE specific for respiratory allergens (e.g., by Phadiatop™) for a group of patients with severe asthma aiming at the determination of asthma phenotypes and for the assessment if cumulative IgE levels specific for respiratory allergens could be a biomarker predicting the success of anti-IgE therapy. By contrast, two studies would have rather suggested the opposite. One study reported that the assessment of pretreatment specific IgE levels is not helpful in predicting the outcome of anti-IgE treatment (47) and a second even suggested that Omalizumab treatment is also effective in non-atopic asthma (48). Moreover, there is growing interest for specific IgE in response prediction also in other settings, such as in atopic dermatitis patients treated with Dupilumab, where Malassezia specific-IgE seems to be associated with the development of Dupilumab-induced facial redness dermatitis (60). However, complex dynamics, possibly involving eosinophils (61), are thought to play a role in the latter.

Other studies suggested that determination of total IgE levels maybe useful to predict efficacy of anti-IgE treatement (42, 44) and a *post-hoc* analysis of poly- versus monosensitized subjects did not provide unequivocal results regarding sensitization status (45).

We hypothesized that the measurement of cumulative IgE levels specific for respiratory allergens by Phadiatop™ testing may be useful for asthma phenotyping and in particular to identify patients with allergen-triggered asthma to choose IgE-targeted strategies for the 5th stage of therapy for severe asthma. The use of the Phadiatop™ test for this purpose seemed justified to us by the fact that it detects increased levels of specific IgE to respiratory allergens to suggest respiratory allergen exposure as possible asthma trigger factor.

It is common practice among clinicians to use total IgE levels as a biomarker that can be used to determine the allergic phenotype of asthma. However, it often turns out, and the results of our study support this assumption, that total IgE measurements are not sufficient and additional testing for allergen-specific IgE is required, which ultimately is more costly than using the Phadiatop™ as first line screening test. Skin tests with allergens are in principle cheaper than *in vitro* determination of specific IgE, but only a limited number of allergen sources can be tested and the procedure is time consuming and may require multiple visits of the patient. Furthermore, *in vivo* tests can be performed in controlled asthma patients and a FEV1 level > 70%, but may be risky in patients suffering from severe asthma. Obtaining a profound and complete anamnesis regarding allergy status is not always possible in routine clinical practice and needs to be linked with a deep diagnostic work-up regarding allergy by targeted allergy diagnosis selecting the serological tests for measuring allergen-specific IgE and provocation with the correct allergen extracts according to empiric knowledge. Since Phadiatop™ is a simple and inexpensive biomarker for IgE sensitization to respiratory allergens the selection of skin tests with allergens and/or laboratory tests determining the level of specific IgE to preselected allergens, according to the allergic anamnesis data may be reduced or even avoided. Similar results may be achieved with allergen chips containing a large panel of micro-arrayed allergens but the costs for chip testing are currently considerably higher (17).

Our study indicates that Phadiatop™ testing may indeed be useful to identify patients who are suitable for IgE-targeted molecular therapy and demonstrates that patients with allergen-specific levels over a certain threshold benefit more than those below this threshold. The study also showed that the level of total IgE did not depend on the positive/negative allergic anamnesis (p = 0.218) and the result of skin tests (p = 0.1), which has been also reported by other researchers (23, 26, 27, 31). Statistically significant differences in the level of total IgE were obtained depending on the result of specific IgE (p = 0.047), but with wide confidence intervals, which does not allow relying on the accuracy of the method. Cumulative IgE levels specific for respiratory allergens as measured by Phadiatop™ were statistically significantly higher in groups of patients with positive allergic anamnesis, positive skin tests and positive sIgE results than in groups with no allergic anamnesis, negative skin tests and negative sIgE results (**p <0.001, ). The increase in the total IgE level was associated with different phenotypes of the asthma, whereas cumulative IgE levels specific for respiratory allergens were significantly higher in patients with an allergic component in the pathogenesis of asthma (J45.0 and J45.8).

It is generally considered that a Phadiatop™ test result ≥ 0.35 PAU/l is positive. However, in practice, it is often observed that the patient does not have a clinically relevant reaction with a weakly positive result in the Phadiatop™ test. For our set of patients a cut-off point of 1.53 PAU/l was obtained (Se 62.5%, Spec 86.1%). Due to the low sensitivity and high specificity, if the result is less than 1.53 PAU/l in the patient it can be predicted that the patient will not have clinically relevant sensitization. The cut-off level found by us is higher than that of Zeng *et al.,* 2018 (0.53, Se 89.4, Spec 97.5%) (30) which is possibly due to differences in the study groups and the allergen molecules responsible for sensitization in the two study populations. In fact, house dust mite allergy is very common in the Chinese population (62) whereas it is rare in allergic subjects in Russia due to climate differences (63). Accordingly, it is likely that cut-off levels for specific IgE levels determined by PhadiatopTM test as biomarker may vary in subjects with different IgE sensitization profiles.

Comparison of the efficacy parameters of the Phadiatop™ test when used as isolated biomarker with the collection of allergic anamnesis, skin tests and the determination of specific IgE does not allow us to conclude that the Phadiatop™ test has a clear advantage for asthma phenotyping. However, the characteristics of the combination of the Phadiatop™ test with allergic anamnesis were superior to both allergic anamnesis and standard methods separate and in combination. Thus, Phadiatop™, in combination with the collection of an allergic anamnesis, can be used instead of cumber-some standard skin testing and hypothesis-driven determination of specific IgE.

Indeed, response prediction to Omalizumab is challenging. This is true also in the setting of chronic spontaneous urticaria, where low total IgE is a strong predictor of poor response (64, 65), but specific IgE levels against a number of autoallergens (e.g., Fc epsilon R1, IL-24 and others) also may have some role in response prediction - although the evidence at the moment is conflicting (66). To evaluate the effectiveness of the proposed method for selecting patients for molecular therapy targeting IgE, we conducted a comparative analysis of treatment results with an anti-IgE drug in two groups of patients, with an initial value of the Phadiatop™ test < 1.53 PAU/l and ≥ 1.53 PAU/l. The dynamics of Omalizumab therapy results in the group with initial Phadiatop™ ≥ 1.53 PAU/l was superior to the results of therapy in the group with initial Phadiatop™ ≥ 0.35 and < 1.53 in terms of the level of asthma control (the proportion of uncontrolled asthma and an increase in AСT scores), a decrease in the number of exacerbations of asthma, improvement of the quality of life (according to AQLQ questionnaire).

It has been suggested that typing of asthma according to underlying trigger factors may be useful for guiding newly emerging forms of molecular and personalized treatment of patients suffering from asthma. The limitations of our study are associated with the retrospective analysis and the absence of a control group comprisig patients with non-allergic severe asthma treated with Omalizumab. Furthermore, we were able to analyze only a relatively small number of patients with severe asthma for whom all diagnostic methods were performed and who were only treated with Omalizumab. However, it must be acknowledged that severe asthma patients account only for approximately 5% to 10% of patients with asthma, which makes it very difficult to find them in real clinical practice (67). Nevertheless, our study demonstrated clearly that patients with IgE levels above 1.53 PAU/l achieved better asthma control with molecular therapy targeting IgE than patients with allergen-specific IgE levels below 1.53 PAU/l. Our study thus demonstrates that it may be possible to use Phadiatop™ as biomarker to determine cut-off levels of allergen-specific IgE above which clinical effects of IgE-targeted molecular therapy are better than in severe asthma patients with allergen-specific IgE levels below the cut-off. Therefore, the measurement of cumulative IgE levels with a screening test comprising respiratory allergens (i.e., Phadiatop™) seems superior to the measurement of total IgE levels and classical hypothesis-driven methods for allergy diagnosis for identifying patients with severe allergic asthma and can enhance the clinical efficacy of IgE-targeted asthma therapy. Our results were obtained in a single region pilot study with a relatively simple and crude test for measuring allergen-specific IgE. Accordingly, further studies involving larger numbers of patients with severe asthma and more sophisticated tests (e.g., chips containing micro-arrayed allergen molecules) for measuring allergen-specific IgE will be needed to further investigate the usefulness of measuring allergen-specific IgE as biomarker for prescription and prediction of efficacy of IgE-targeted therapies in asthma.

## Data availability statement

The raw data supporting the conclusions of this article will be made available by the authors, without undue reservation.

## Ethics statement

The studies involving human participants were reviewed and approved by Ethics Committee of the Ural State Medical University. The patients/participants provided their written informed consent to participate in this study.

## Author contributions

Conceptualization, VN, EB, DK, AK and RV. Methodology, VN, DK. Formal analysis, VN, EB, DK, and RV. Investigation, VN, EB, DK, and RV. Resources, VN, EB, DK, and RV. Data curation, VN, EB, DK, and RV. Writing — original draft preparation, VN, EB, DK. Writing — review and editing, KN, PE, and RV. Visualization, VN, EB, DK, KN. All authors have read and agreed to the published version of the manuscript.

## Funding

RV is a recipient of a Megagrant of the Government of the Russian Federation, grant number 14.W03.31.0024. This work was partially supported by the “Danube Allergy Research Cluster” Program of the Lower Austria (330950005).

## Conflict of interest

RV has received research grants from HVD Life-Sciences, Vienna, Austria, and WORG Pharmaceuticals, Hangzhou, China and from Viravaxx AG, Vienna, Austria. Furthermore, he serves as consultant for WORG and Viravaxx AG.

The remaining authors declare that the research was conducted in the absence of any commercial or financial relationships that could be constructed as a potential conflict of interest.

## Publisher’s note

All claims expressed in this article are solely those of the authors and do not necessarily represent those of their affiliated organizations, or those of the publisher, the editors and the reviewers. Any product that may be evaluated in this article, or claim that may be made by its manufacturer, is not guaranteed or endorsed by the publisher.
